# The microRNAs miR-200b-3p and miR-429-5p target the LIMK1/CFL1 pathway to inhibit growth and motility of breast cancer cells

**DOI:** 10.18632/oncotarget.19205

**Published:** 2017-07-12

**Authors:** Dengfeng Li, Hong Wang, Hongming Song, Hui Xu, Bingkun Zhao, Chenyang Wu, Jiashu Hu, Tianqi Wu, Dan Xie, Junyong Zhao, Qiang Shen, Lin Fang

**Affiliations:** ^1^ Department of Thyroid and Breast, Division of General Surgery, Shanghai Tenth People’s Hospital, Tongji University School of Medicine, Shanghai 200072, People’s Republic of China; ^2^ Department of General Surgery, Zhongshan Hospital, Fudan University, Shanghai 200032, People’s Republic of China; ^3^ Department of Clinical Cancer Prevention, Division of Cancer Prevention and Population Science, The University of Texas M.D. Anderson Cancer Center, Houston, Texas 77030, United States

**Keywords:** miR-200b-3p, miR-429-5p, triple-negative breast cancer, proliferation, cell motility

## Abstract

Triple-negative breast cancer (TNBC) has the worst prognosis of all subtypes of breast cancer (BC), with limited options for conventional therapy and no targeted therapies. MicroRNAs (miRNAs) are small noncoding RNAs that negatively regulate gene expression. In this study, we aimed to determine whether two members of the miR-200 family, miR-200b-3p and miR-429-5p, are involved in BC cell proliferation and motility and to elucidate their target genes and pathways. We performed a meta-analysis that reveals down-regulated expression of miR-200b-3p and miR-429-5p in BC tissues and cell lines, consistent with a lower expression of miR-200b-3p and miR-429-5p in MDA-MB-231 and HCC1937 cells than in MCF-7 and MCF-10 cells. Overexpression of miR-200b-3p and miR-429-5p significantly inhibited the proliferation, migration, and invasion of TNBC cells; suppressed the expression of markers for proliferation and metastasis in TNBC cells. We next demonstrated that LIM domain kinase 1 (*LIMK1*) is a direct target gene of miR-200b-3p and miR-429-5p. Inhibition of *LIMK1* reduced the expression and phosphorylation of cofilin 1 (*CFL1*), which polymerizes and depolymerizes F-actin and G-actin to reorganize cellular actin cytoskeleton. In addition, transfection with mimics for miR-200b-3p and miR-429-5p arrested G2/M and G0/G1 cell cycles respectively, suppressed the expression of the cell cycle–related complexes, cyclin D1/CDK4/CDK6 and cyclin E1/CDK2, in TNBC cells. In conclusion, miR-200b-3p and miR-429-5p suppress proliferation, migration, and invasion in TNBC cells, via the LIMK1/CFL1 pathway. These results provide insight into how specific miRNAs regulate TNBC progression and suggest that the LIMK1/CFL1 pathway is a therapeutic target for treating TNBC.

## INTRODUCTION

Breast cancer (BC) is a major global health burden, with more than 1 million new cases diagnosed yearly [1] . Approximately 70% to 80% of BCs express hormone receptors—estrogen receptors, progesterone receptors, or human epidermal growth factor receptor 2 on the cell surface, but about 15% to 20% of BCs are the triple-negative BC (TNBC) subtype, which expresses no hormone receptors [1, 2]. Although endocrine therapy is a major treatment option for hormone receptor-positive BCs, no targeted therapies are available for TNBC, which tends to be resistant to chemo- and radiotherapy [1, 3]. Therefore, patients with TNBC have higher relapse and mortality rates and worse outcomes than do patients with hormone receptor-positive BC [4]. Thus, understanding the mechanisms underlying TNBC progression is important for developing future targeted therapies.

MicroRNAs (miRNAs) are a large group of small, noncoding, single-stranded RNAs that modulate target mRNAs to inhibit protein translation. MiRNAs can function as tumor suppressors or onco-miRs [5, 6]. MiR-200b-3p and miR-429-5p belong to the miR-200 family, which contains 5 miRNAs, including 2 miRNA gene clusters (mir-200b/a/429 and mir-200c/141) [6]. The miR-200 family members are associated with regulating epithelial-to-mesenchymal transition (EMT) and mesenchymal-to-epithelial transition, cancer cell proliferation, and drug resistance. Loss of expression of the miR-200 family in breast cells is linked with initiation of EMT and with cancer invasion [7]. Researchers also reported that miR-200b-3p inhibited migration and invasion of BC cells by regulating ezrin-radixin-moesin protein expression [8]. MiR-200b-3p also suppressed BC cell growth and induced apoptosis in BC cells by regulating the nuclear factor-kappa B pathway via the inhibitor of nuclear factor-kappa B kinase subunit beta (IKBKB/IKK-β) [9]. Similarly, miR-429-5p inhibited migration and invasion of BC cells by suppressing the expression of zinc finger E-box binding homeobox 1 (*ZEB1*) and CRK-like proto-oncogene, adaptor protein (*CRKL*) [10] and induced apoptosis in BC cells by regulating expression of X-linked inhibitor of apoptosis (*XIAP*) [11]. However, roles of miR-200b-3p and miR-429-5p in TNBC progression in particular are less well understood, and whether other direct targets for miR-200b-3p and miR-429-5p are important for TNBC progression remains unknown.

LIM domain kinase 1 (LIMK1) is a serine/threonine kinase that phosphorylates and inactivates cofilin (CFL), an actin-binding factor, to promote actin polymerization. *LIMK1* is involved in regulating cell proliferation and invasion and is activated and regulated by the Rho family of small GTPases. Members of the CFL family serve as the substrates for LIMK1. LIMK1 is required for inactivation of CFL1, an essential factor for promoting local F-actin stability and the formation and maturation of functional invadopodia [12]. LIM domain kinases are also required for cell invasion; they promote the formation of invasive paths in collagen-rich environments during cancer cell migration [13]. However, whether specific miRNAs regulate the expression of LIMK1 and thereby modulate TNBC cell motility and tumor progression is not well understood.

The purpose of this study was to determine the mechanisms that regulate breast cancer progression and metastasis. We hypothesized that miR-200b-3p and miR-429-5p are key miRNAs regulating TNBC proliferation, migration, and invasion in TNBC cells. As a first step, we determined via a meta-analysis of publications included in multiple publicly available databases lines [14–24] that expression of miR-200b-3p and miR-429-5p was lower in BC tissue and cell lines than in normal breast tissues and mammary epithelial cells. We then detected the expression of miR-200b-3p and miR-429-5p in MDA-MB-231, HCC1937 and MCF-7 cells, in comparison with MCF-10A, an immortal mammary epithelial cell line. We found that the expression of miR-200b-3p and miR-429-5p was lower than in MCF-7 and MCF-10A cells. Therefore we focus on MDA-MB-231 and HCC1937 cells. We then determined that miR-200b-3p and miR-429-5p target the *LIMK1* gene and inhibit the LIMK1/CFL1 pathway. Gain-of-function assessments validated a tumor-suppressing role for miR-200b-3p and miR-429-5p in TNBC cells. Our findings deepen our understanding of TNBC progression and provide a rational basis for developing targeted strategies to enhance miR-200b-3p and miR-429-5p expression or block the LIMK1/CFL1 pathway for treating TNBC.

## RESULTS

### Expression of miR-200b-3p and miR-429-5p in BC cells

**Figure 1:**
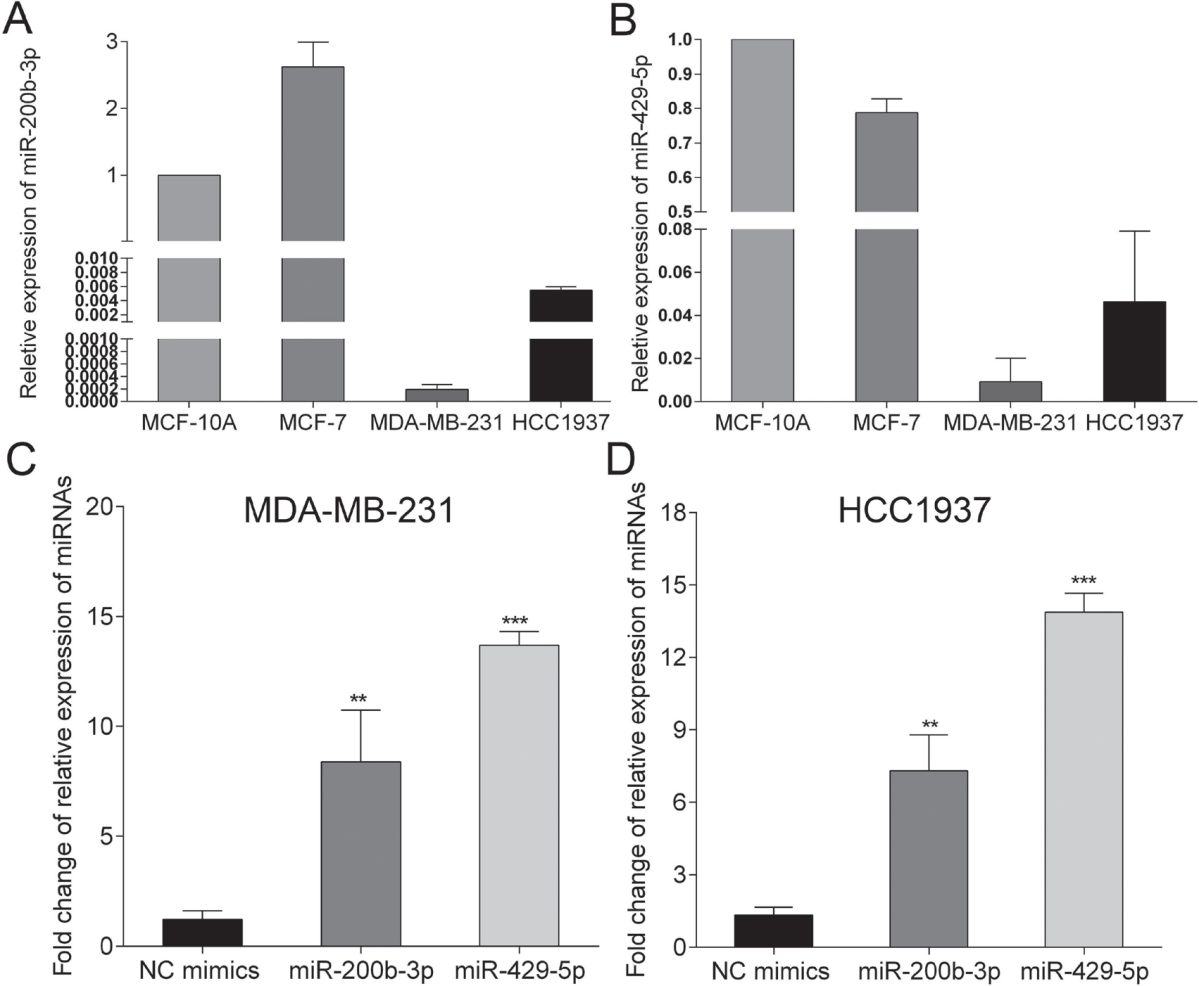
Expression of miR-200b-3p and miR-429-5p in breast cancer cell lines (**A**, **B**) expression of miR-200b-3p and miR-429-5p were lower in MDA-MB-231 and HCC1937 breast cancer cells, compared to MCF-7 and MCF-10A cells. (**C**, **D**) transfection of miR-200b-3p and miR-429-5p mimics increased the expression of miR-200b-3p and miR-429-5p in MDA-MB-231 and HCC1937 breast cancer cells.

We started with the determination of expression of miR-200b-3p and miR-429-5p in BC tissue and cell lines via a meta-analysis of publications included in publicly available databases. Expression of miR-200b-3p and miR-429-5p was lower in BC tissue and BC cell lines than in normal breast tissue and mammary epithelial cells (Supplementary Table 1). We next determined the expression of miR-200b-3p and miR-429-5p in MDA-MB-231, HCC1937 and MCF-7 cells, in comparison with MCF-10A, an immortal mammary epithelial cell line. We found that the expression of miR-200b-3p and miR-429-5p was lowest in MDA-MB-231 cells, lower than in MCF-7 and MCF-10A cells (Figure 1A and 1B). Therefore we chose to focus on MDA-MB-231 and HCC1937 cells triple-negative BC cells. After transferring miR-200b-3p and miR-429-5p mimics, the expression of miR-200b-3p and miR-429-5p significantly increased (Figure 1C), suggesting that these mimics could upregulate the expression of miR-200b-3p and miR-429-5p in MDA-MB-231 and HCC1937 cells.

### Enhancement of miR-200b-3p and miR-429-5p expression inhibits proliferation of TNBC cells

**Figure 2:**
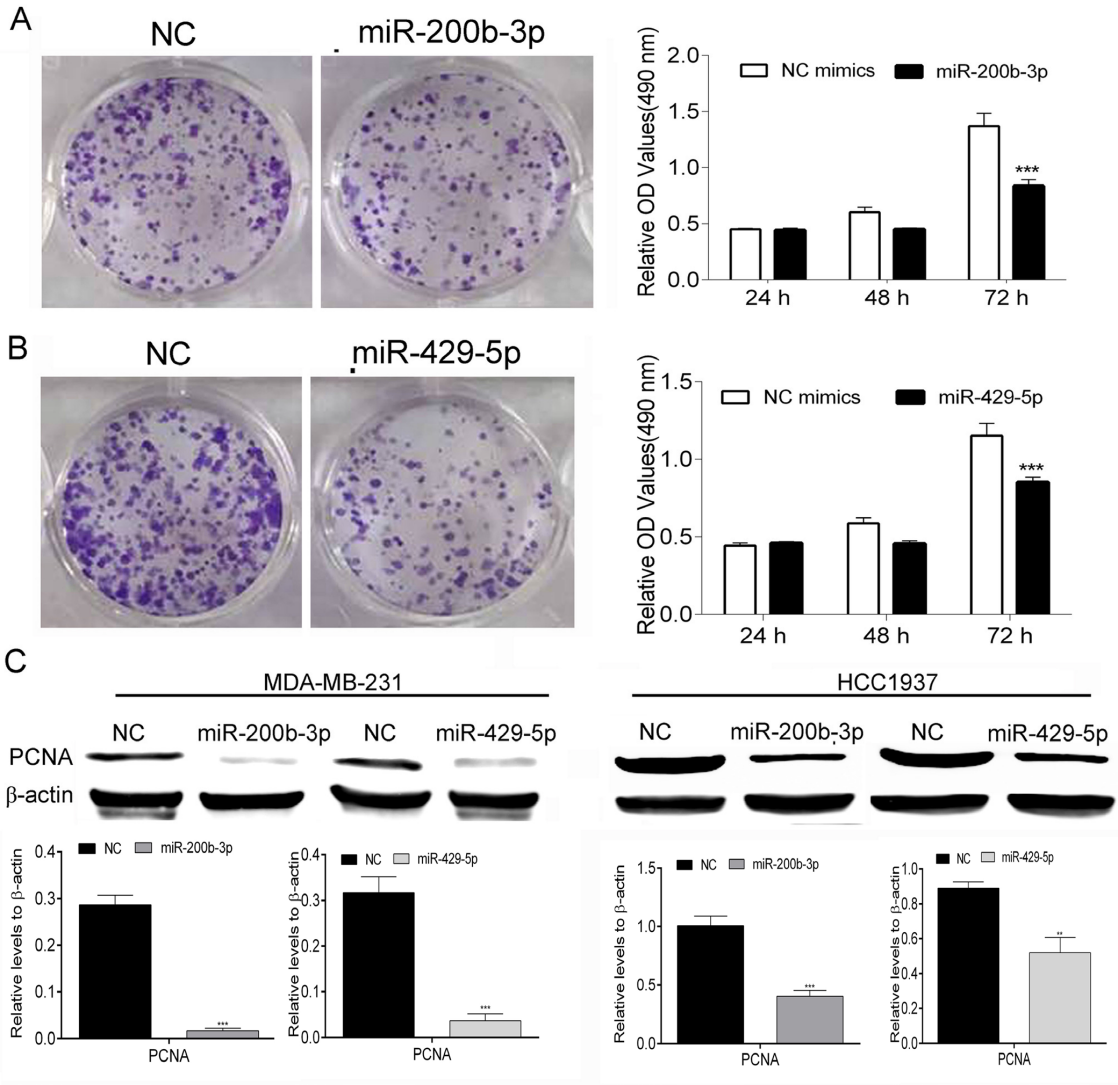
MiR-200b-3p and miR-429-5p suppressed proliferation of TNBC cells (**A**) Representative colony-formation assays and results of MTT assays showing that transfection with miR-200b-3p mimics suppressed the proliferation of MDA-MB-231 TNBC cells. (**B**) Representative colony-formation assays and results of MTT assays showing that transfection with miR-200b-3p mimics suppressed the proliferation of TNBC cells (One-way ANOVA analysis of variance; miR-200b-3p and miR-420-5p vs NC, *p* < 0.001). (**C**) Representative Western blots showing that miR-200b-3p and miR-429-5p inhibited the expression of PCNA in MDA-MB-231 and HCC1937 TNBC cells. NC, negative control; OD, optical density. Three independent experiments were performed. Numbers of colony or gray scale images of each assay were counted or scanned and presented as a bar chart in which the vertical axis is the mean ± SD. *** indicates statistical differences at *p* < 0.001.

We performed colony-formation and MTT assays to evaluate the effect of overexpression of miR-200b-3p or miR-429-5p on the proliferation of MDA-MB-231 TNBC cells. We found that transfection with mimics of miR-200b-3p and miR-429-5p decreased MDA-MB-231 cells’ colony-forming ability from the levels observed in cells transfected with NC mimics. The MTT assays demonstrated that transfection with miR-200b-3p and miR-429-5p mimics inhibited the growth of MDA-MB-231 cells in a time-dependent manner notably (*p* < 0.05) (Figure 2A and 2B). These changes were consistent with our observation of lower protein expression of PCNA, a proliferation marker, in miR-200b-3p– and miR-429-5p–transfected MDA-MB-231 cells then in identically transfected HCC1937 cells (Figure 2C). These results suggested that miR-200b-3p and miR-429-5p regulate the proliferation of BC cells.

### Overexpression of miR-200b-3p and miR-429-5p inhibits the motility of BC cells

**Figure 3:**
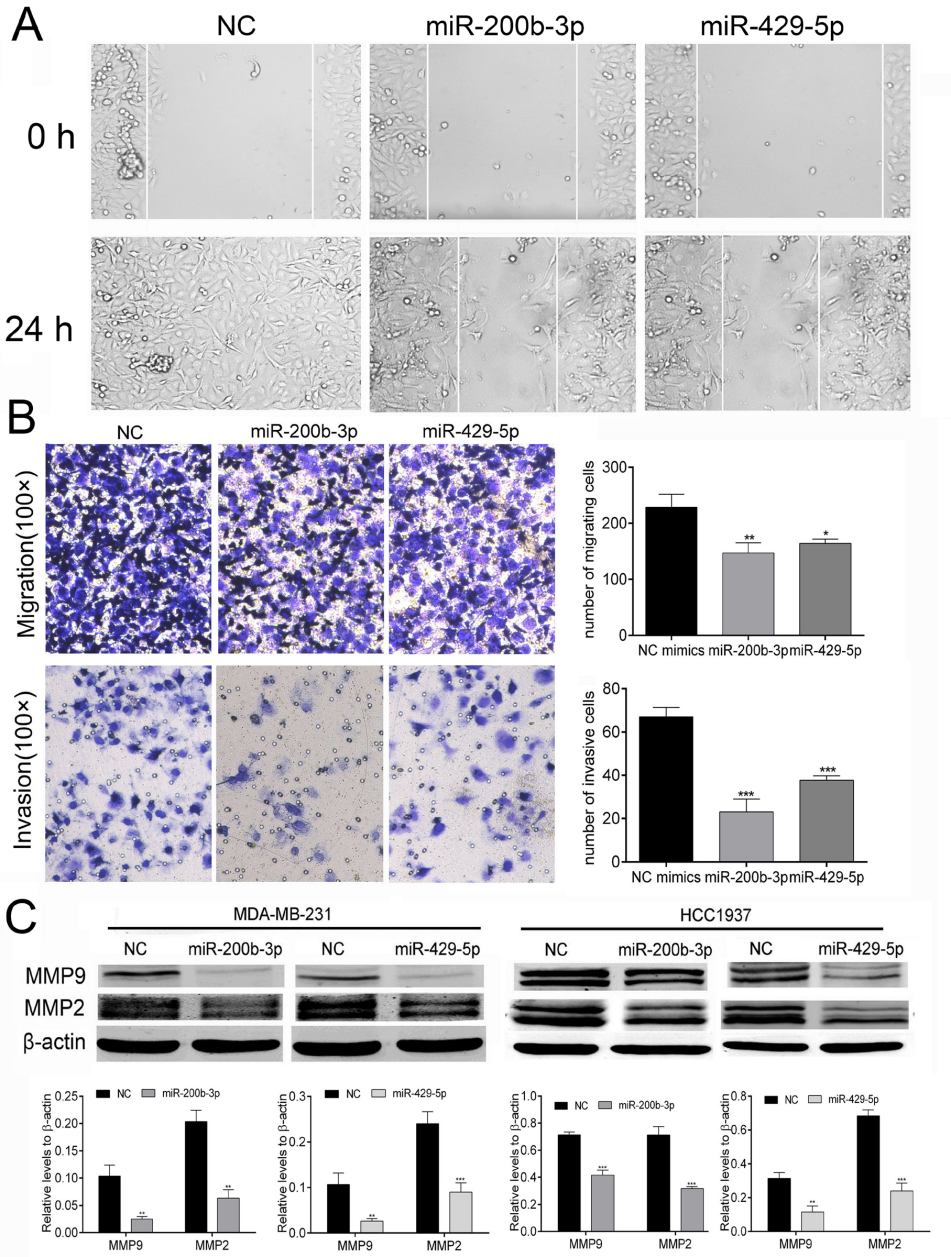
MiR-200b-3p and miR-429-5p suppressed the mobility of TNBC cells (**A**) Representative images for wound healing assessments. (**B**) Representative images from Transwell assays (migration and invasion) showing that transfection with miR-200b-3p and miR-429-5p mimics suppressed the migration and invasion ability of MDA-MB-231 TNBC cells (100 fold). Quantification of data is shown as bar graphs. (**C**) Representative Western blots showing that transfection with miR-200b-3p and miR-429-5p suppressed expression of MMP2 and MMP9 in both MDA-MB-231 and HCC1937 TNBC cells. Quantification of data is shown as bar graphs. NC, negative control. Three independent experiments were performed. Numbers of invading cells or gray scale images of each assay were counted or scanned and presented as a bar chart in which the vertical axis is the mean ± SD. ** indicates statistical differences at *p* < 0.01 and *** indicates statistical differences at *p* < 0.001.

We performed wound-healing assay and transwell assay to test whether transfection with mimics of miR-200b-3p or miR-429-5p affected the ability of MDA-MB-231 TNBC cells to migrate and invade. As shown in Figure 3A and Figure 3B, overexpression of miR-200b-3p or miR-429-5p significantly inhibited the migration and invasion of MDA-MB-231 cells. We next measured the expression levels of the matrix metalloproteinases MMP2 and MMP9 in MDA-MB-231 and HCC1937 cells. MMP2 and MMP9 are enzymes important for metastatic cancer cell to invade the basement membrane, and therefore are indicators of metastasis. MMP2 is highly expressed in cancer tissues and high expression of active MMP2 is associated with risk of metastasis [25]. MMP-9 expression is also significantly correlated with BC grades and overall survival [26]. We therefore chose MMP2 and MMP9 as the representative biomarkers to reflect migration and invasion of metastatic BC cells. As shown in Figure 3C, overexpression of miR-200b-3p and miR-429-5p significantly reduced the expression of MMP2 and MMP9 in both MDA-MB-231 and HCC1937 TNBC cells. These results suggested that gain of function of miR-200b-3p or miR-429-5p inhibits the motility of BC cells.

### The LIMK1/CFL1 pathway is regulated by miR-200b-3p and miR-429-5p in BC cells

**Figure 4:**
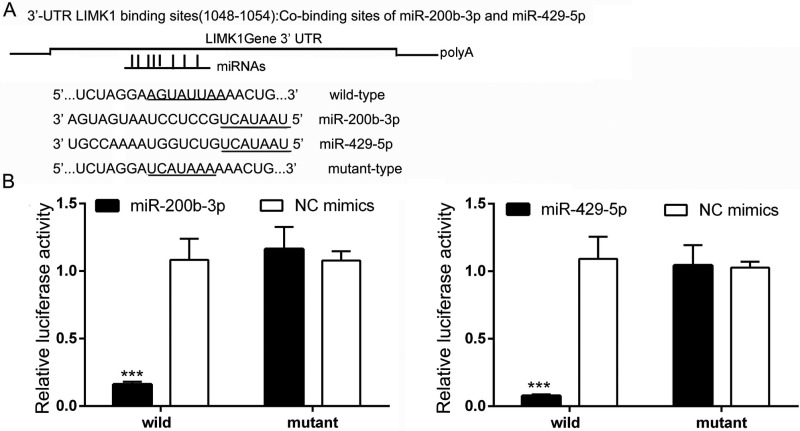
*LIMK1* is a direct target gene of miR-200b-3p and miR-429-5p in TNBC cells (**A**) The binding sites of miR-200b-3p and miR-429-5p and the 3′-UTR of *LIMK1* (wild-type and mutant). (**B**) Bar graphs showing that transfection with miR-200b-3p or miR-429-5p significantly suppressed luciferase activity in wild-type in HEK 293T cells (Student *t* test; *p* < 0.05). NC, negative control. Three independent experiments were performed. Relative luciferase activity were recorded and presented as a bar chart in which the vertical axis is the mean ± SD. *** indicates statistical differences at *p* < 0.001.

We next identified the target genes of miR-200b-3p and miR-429-5p. A search of the TargetScan database revealed that the binding sites for miR-200b-3p and miR-429-5p are located at 1048 to 1054 bp downstream from the 5′ end of *LIMK1*, suggesting that *LIMK1* is a potential target gene of miR-200b-3p and miR-429-5p. We therefore constructed plasmids containing wild-type and mutant binding sites for miR-200b-3p and miR-429-5p (Figure 4A). The plasmids were transfected into HEK 293T cells to verify whether miR-200b-3p or miR-429-5p targeted the 3′-UTR of *LIMK1*. As shown in Figure 4B, dual-luciferase activity at the *LIMK1* wild-type binding sites was significantly lower in miR-200b-3p– and miR-429-5p–treated HEK 293T cells than in NC-treated cells. In addition, when the *LIMK1* binding sites were mutated, miR-200b-3p and miR-429-5p no longer inhibited luciferase activity. These results demonstrated that *LIMK1* is a direct target gene of both miR-200b-3p and miR-429-5p.

**Figure 5:**
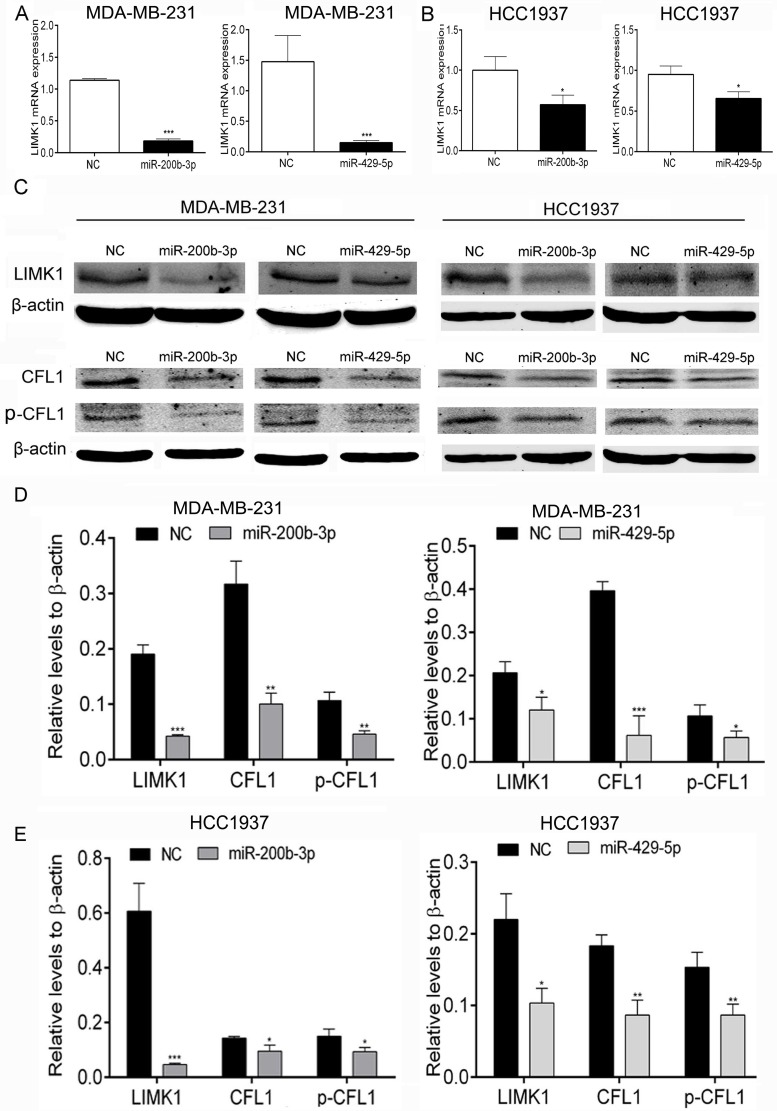
MiR-200b-3p and miR-429-5p suppressed expression of LIMK1 and its substrate CFL1 in TNBC cells (**A**) and (**B**), Bar graphs showing results of dual-luciferase reporter assays for *LIMK1* mRNA expression. Transfection with miR-200b-3p or miR-429-5p decreased the level of *LIMK1* mRNA expression in MDA-MB-231 and HCC1937 TNBC cells (Student *t* test; *p* < 0.001). (**C**–**E**), Representative Western blots showing that transfection with miR-200b-3p or miR-429-5p suppressed the LIMK1/CFL1 pathway in MDA-MB-231 and HCC1937 cells. NC, negative control. Three independent experiments were performed. Relative mRNA levels or gray scale images of each assay were quantitated or scanned and presented as a bar chart in which the vertical axis is the mean ± SD. * indicates statistical differences at *p* < 0.05, ** indicates statistical differences at *p* < 0.01, and *** indicates statistical differences at *p* < 0.001.

To further validate these results, we performed a dual-luciferase reporter assay using MDA-MB-231 and HCC1937 cells. We found that transfection with miR-200b-3p and miR-429-5p mimics significantly inhibited the expression of *LIMK1* mRNA in MDA-MB-231 and HCC1937 TNBC cells, as shown in Figure 5A–5B. At the protein level, LIMK1 transcription was markedly lower in MDA-MB-231 and HCC1937 cells transfected with miR-200b-3p or miR-429-5p mimics than in cells transfected with NC mimics (Figure 5C, 5D and 5E). We also found that miR-200b-3p and miR-429-5p transfection significantly reduced CFL1 protein expression and CFL1 phosphorylation in both TNBC cell lines (Figure 5C, 5D and 5E). These results suggested that miR-200b-3p and miR-429-5p regulate the expression of *LIMK1* and inhibit the LIMK1/CFL1 pathway in BC cells.

### MiR-200b-3p and miR-429-5p regulate cell-cycle progression of BC cells in different phases

**Figure 6:**
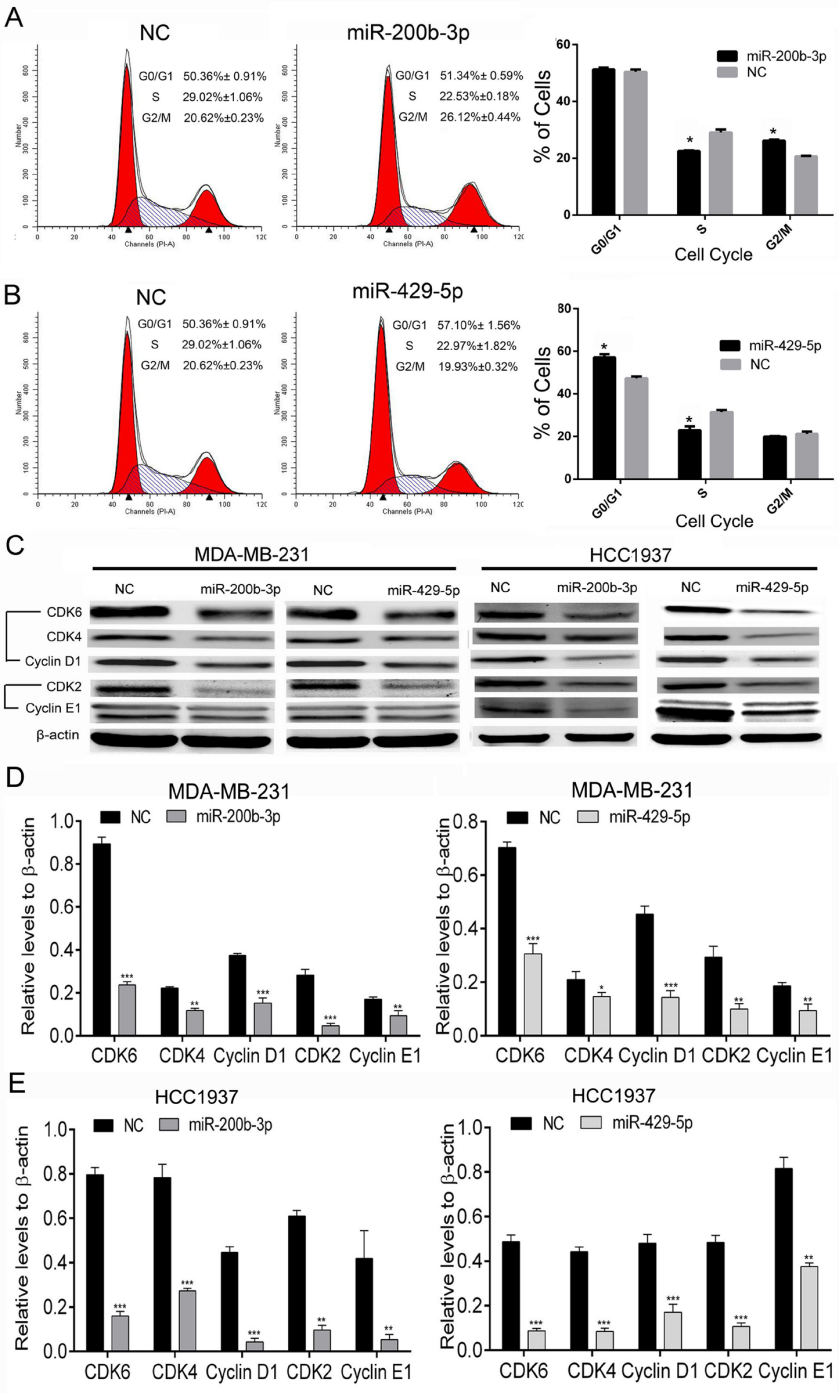
Gain of function of miR-200b-3p or miR-429-5p arrests cell-cycle progression in TNBC cells (**A**) Flow cytometry results demonstrating that miR-200b-3p arrested more MDA-MB-231 TNBC cells than negative control (NC) cells at G2/M phase (Student *t* test; *p* < 0.05). (**B**) Flow cytometry results demonstrating that miR-429-5p arrested more MDA-MB-231 cells than NC cells at G0/G1 phase (Student *t* test; *p* < 0.05). (**C**–**E**) Representative Western blots showing that miR-200b-3p and miR-429-5p suppressed CDK4/CDK6/cyclin D1 and CDK2/cyclin E1 protein expression in MDA-MB-231 and HCC1937 TNBC cells. Three independent experiments were performed. % of cells or gray scale images of each assay were quantitated or scanned and presented as a bar chart in which the vertical axis is the mean ± SD. * indicates statistical differences at *p* < 0.05, ** indicates statistical differences at *p* < 0.01, and *** indicates statistical differences at *p* < 0.001.

We next examined whether gain of function of miR-200b-3p and miR-429-5p affects cell-cycle progression. The miR-200b-3p and miR-429-5p mimics were transfected into MDA-MB-231 cells, which were then analyzed using flow cytometry. As shown in Figure 6A, transfection with miR-200b-3p mimics arrested significantly more MDA-MB-231 cells at G2/M phase than did transfection with NC mimics. However, transfection with miR-429-5p mimics arrested more MDA-MB-231 cells at G0/G1 phase than did transfection with NC mimics, as shown in Figure 6B. We then detected cell cycle–related proteins in TNBC cells using Western blotting. We found that both miR-200b-3p and miR-429-5p mimics inhibited the expression of cyclin D1/CDK4/CDK6 and cyclin E1/CDK2 in MDA-MB-231 and HCC1937 cells (Figure 6C, 6D and 6E). These results suggested that miR-200b-3p and miR-429-5p regulate cell-cycle progression in BC cells.

### Protein-protein interaction network among miR-200b-3p, miR-429-5p and LIMK1-CFL1 pathway

**Figure 7:**
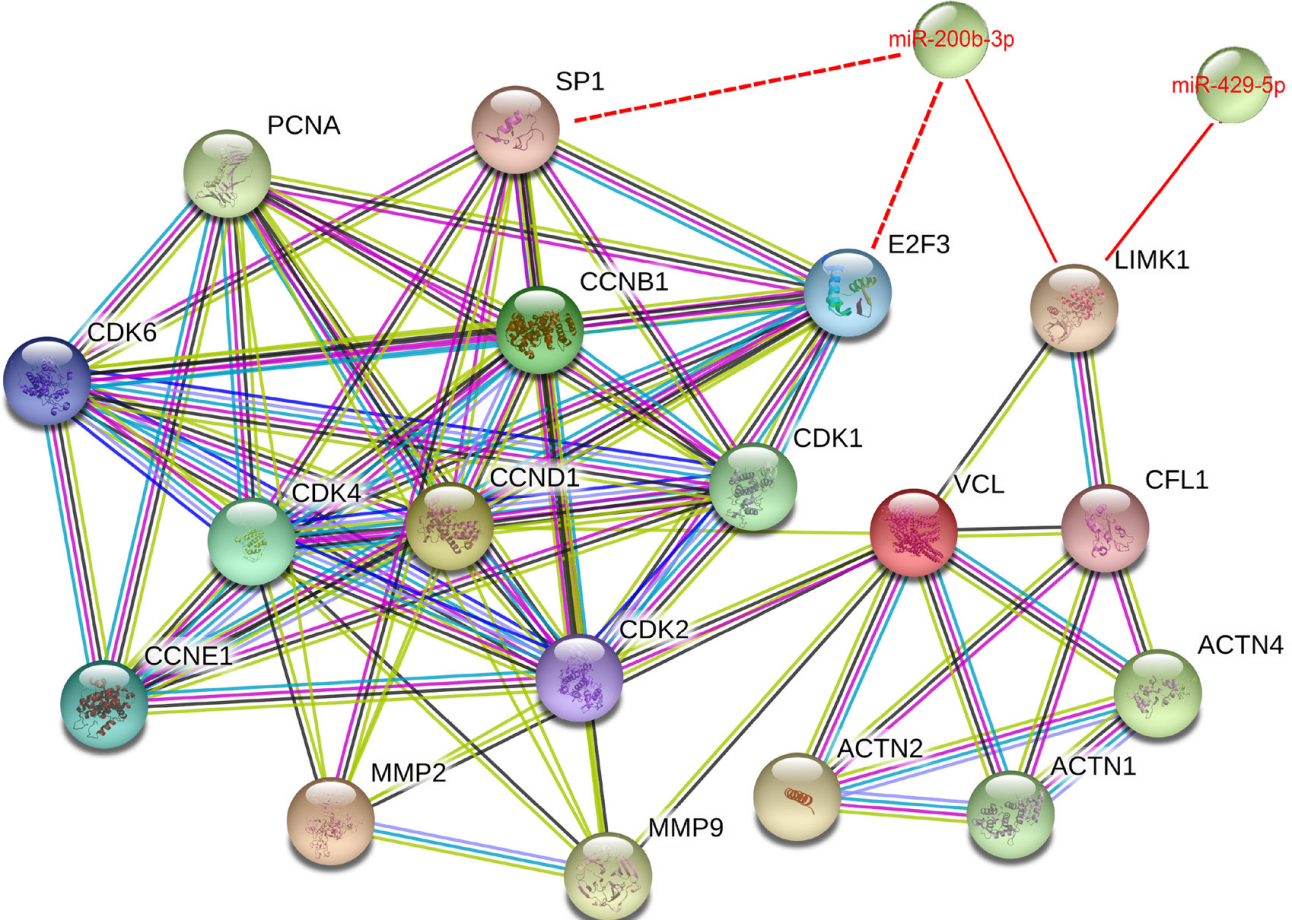
Protein-protein interactions among miR-200b-3p and miR-429-5p regulated proteins The protein-protein interactions of how miR-200b-3p and miR-429-5p regulates LIMK1-CFL1 pathway and proteins involved in cell proliferation and metastasis process, obtained using STRING program at https://string-db.org/.

We then performed an interaction pathway analysis using the STRING database (http://string-db.org/). The analysis showed the potentially regulated interacting proteins on LIMK1-CFL1 pathway and the proteins involved in cell proliferation and metastasis process by miR-200b-3p and miR-429-5p (Figure 7). Both miRNAs regulate the LIMK1-CFL1 pathway, through ACTN1-VCL (vinculin, F-actin binding proteins) to regulate cyclin D1/CDK4/CDK6, cyclin E1/CDK2 and MMP2, and MMP9 proteins. Vinculin (VCL) is a key protein to connect actin cytoskeleton and cell cycle-related complexes. It was reported that ERα suppresses the movement of BC cells by up-regulating VCL [27]. In another study, nuclear calcium buffering regulates BC cell motility, likely due to enhanced VCL expression [28]. The analysis also showed other potential targets of miR-200b-3p regulating CDK family members, such as SP1 and E2F3. The insert shows the context menu available for all STRING proteins and links between protein interaction network, as colored by evidence type (yellow line-text mining, grey line-homology, light blue-database, dark blue line-cooccurrence, pink line-means experiments, and black line-co-expression. Color legend can be found on STRING website).

## DISCUSSION

In this study, our meta-analysis of 11 publicly available databases demonstrated that the expression of miR-200b-3p and miR-429-5p was markedly lower in BC cells and BC tissues than in normal breast cells and tissues (Supplementary Table 1). We further validated the expression of miR-200b-3p and miR-429-5p in different BC cell lines that showed lower expression of miR-200b-3p and miR-429-5p in MDA-MB-231 and HCC1937 cells than that in MCF-7 and MCF-10A cells (Figure 1). We therefore used miR-200b-3p and miR-429-5p mimics as molecular tools for further experiments in MDA-MB-231 and HCC1937 cell lines. We determined that miR-200b-3p and miR-429-5p target *LIMK1* and inhibit the LIMK1/CFL1 pathway in TNBC. Gain-of-function assessments validated a tumor-suppressing role for miR-200b-3p and miR-429-5p in TNBC cells. These findings further our understanding of TNBC progression and provide a rational basis for strategies to enhance miR-200b-3p and miR-429-5p expression or block the LIMK1/CFL1 pathway for treating TNBC.

Many miRNAs are known to be involved in tumorigenesis, cancer progression, and apoptosis [29]. Researchers reported that miR-200b-3p regulates several different biological processes by targeting several different cancer-related genes. For instance, miR-200b-3p targeting of Sp1 transcription factor (*SP1*) regulates cell proliferation and apoptosis [14], whereas targeting tumor protein p53 binding protein 1 (*53BP1*) and the Rho GDP disassociation inhibitor alpha (RHOGDI) pathway suppresses EMT [15, 30]. MiR-200b-3p was also reported to play a role in BC lymph node metastasis [24]. Furthermore, studies showed that upregulation of miR-200b-3p and miR-190a and downregulation of miR-512-5p may together be associated with better pathologic response to chemotherapy and better outcomes of breast-conserving surgery for patients with TNBC [31]. Several studies found that expression of both miR-200b-3p and miR-429-5p was downregulated in BC tissues [14, 16, 22–24] and BC cell lines [14–21]. Furthermore, miR-429-5p was reported to suppress migration and invasion of BC cells via targeting of *ZEB1* and *CRKL* [10] and to induce apoptosis via targeting of *XIAP* [11]. Researchers also reported that miR-429-5p and miR-200b-3p together inhibited EMT [30]. Our findings validated the roles of miR-200b-3p and miR-429-5p in suppressing TNBC cell migration and invasion. We also found that miR-200b-3p and miR-429-5p dually inhibited expression of PCNA, MMP2, and MMP9, protein biomarkers of cancer-cell proliferation and motility, in MDA-MB-231 and HCC1937 cells.

In the present study, the STRING analysis (Figure 7) was performed to further explore the potential correlation in these protein interactions. VCL plays an important role in transmitting force from cytoplasmic F-actin to membrane-bound integrins [32]. ACTN1, ACTN2 and ACTN4 are proteins that cross-link with F-actin and thought to be a key component for maintaining the intracellular structures [33–35]. Thus, miR-200b-3p and miR-429-5p through targeting LIMK1-CFL1 pathway, impact VCL, ACTN1, ACTN2, and ACTN4 to regulate the expression of PCNA, cyclin D1/CDK4/CDK6, and cyclin E1/CDK2 complex. Our STRING analysis also revealed that miR-200b-3p could target SP1 and E2F3 to regulate cyclin B1/CDK1 [14, 36]. In addition, The STRING analysis showed that miR-200b-3p and miR-429-5p can regulate MMP2 and MMP9 through interaction of LIMK1-CFL1 pathway and VCL.

Taken together, our findings enlarge the repository of target genes for miR-200b-3p and miR-429-5p and provide new insight into the regulatory networks and pathways that promote carcinogenesis in TNBC. Our results demonstrate that both miR-200b-3p and miR-429-5p play a tumor-suppressive role in TNBC via a previously unreported target, *LIMK1*, which is involved in cellular proliferation and motility. *LIMK1* is required for inactivation of *CFL1*, which is an essential factor promoting local F-actin stability and the formation and maturation of functional invadopodia [12]. F-actin regulates the Hippo signaling pathway and the cell cycle [37, 38].

Suppression of cell cycle-modulating proteins such as cyclin D1/CDK4/CDK6 and cyclin E1/CDK2 arrests cells at G0/G1 phase. In this study, however, we found that miR-200b-3p and miR-429-5p arrested MDA-MB-231 cells in different cell-cycle phases: miR-200b-3p induced G2/M-phase arrest, and miR-429-5p induced G0/G1-phase arrest. Also, cyclin D1/CDK4/CDK6 and cyclin E1/CDK2 expression was consistently inhibited by overexpression of miR-200b-3p or miR-429-5p. The reasons for the differences in the cell-cycle phases affected by the 2 studied miRNAs have not yet determined. It was previously reported that miR-200b-3p reduces CDK2 expression, causing G2/M-phase arrest in esophageal squamous cell carcinoma cells [39]. In addition, the same study demonstrated that miR-200b-3p suppresses expression of platelet-activating factor (PAF), suggesting that the ability of miR-200b-3p to arrest cells at G2/M phase depends on the PAF and Wnt/β-catenin signaling pathways. In another study, miR-200b-3p increases percentage of cells in G2/M phase and decreases population in S phase by targeting SP1 [14]. Sp1 was reported to bind to the CDK1 promoter and functions as a CDK1 repressor [40]. It was also reported that miR-200b reverses chemoresistance of docetaxel-resistant lung adenocarcinoma cells by targeting E2F3 through G2/M phase [36]. E2F3 could modulate genes such as CDK1, Aurora-A, and Survivin. Aurora-A has been demonstrated that its activation is required for mitotic entry, centrosome maturation and separation, and G2 to M transition [41]. MiR-200b-3p may therefore affect cell-cycle progression in TNBC cells through similar mechanisms (potential relationship was shown in Figure 7). However, future studies are needed to confirm this hypothesis.

In conclusion, we found lower expression of miR-200b-3p and miR-429-5p in TNBC tissues and cell lines than in normal mammary tissues in meta-analysis. Overexpression of miR-200b-3p and miR-429-5p suppressed proliferation and motility of TNBC cells via the LIMK1/CFL1 pathway. These results demonstrate that miR-200b-3p and miR-429-5p regulate the LIMK1/CFL1 pathway in addition to their previously known targets *SP1*, *53BP1*, and *RHOGDI* in TNBC cells. This information can be used to develop innovative strategies for suppressing the LIMK1/CFL1 pathway by activating miR-200b-3p, miR-429-5p, or both, thereby inhibiting the progression of BC.

## MATERIALS AND METHODS

### Cell culture and transfection

MDA-MB-231 and HCC1937 TNBC cells were purchased from the Chinese Academy of Science at Shanghai (Shanghai, China). HEK 293T human embryonic kidney cells were a gift from the Department of Laboratory Medicine, Shanghai Tenth People’s Hospital. All cells were cultured in Dulbecco’s modified Eagle’s medium (DMEM; Gibco, Grand Island, NY, USA) supplemented with 10% fetal bovine serum (Gibco, Grand Island, NY,) and penicillin-streptomycin (Sigma-Aldrich; Merck KGaA, Darmstadt, Germany) in an incubator at 37°C in 5% CO_2_.

MiR-200b-3p and miR-429-5p mimics and negative-control (NC) mimics were purchased from Ibsbio (Shanghai, China). For transfection, cells (3 × 10^5^/well) were cultured in a 6-well plate until they reached 40% to 50% confluence, and the mimics were transfected with Lipofectamine 2000 (Invitrogen, Carlsbad, CA, USA) according to the manufacturer’s instructions. The concentration of mimics used was 100 nM/L, and the ratio of mimics to Lipofectamine 2000 was 1.25:1.00 (volume). The sequence of the miR-200b-3p mimics was: sense, 5′-UAAUACUGCCUGGUAAUGAUGA-3′; antisense, 5′-AUCAUUACCAGGCAUAUUAUU-3′. The miR-429-5p mimic sequence was: sense, 5′-UAAUACUGUCUGGUAAAACCGU-3′; antisense, 5′-GGUUUUACCAGACAGUAUUAUU-3′. The NC mimic sequence was: sense, 5′-UCACAACCUCCUAGAAAGAGUAGA-3′; antisense, 5′-UACUCUUUCUAGGAGGUUGUGAUU-3′.

### Cell proliferation assays

MDA-MB-231 cells were seeded in a 6-well plate and allowed to reach 40% to 50% confluence. The cells were then transfected with 100 nM miR-200b-3p mimics, miR-429-5p mimics, or NC mimics for 24 h. Single-cell suspensions of the transfected cells were prepared, and the cells were replated in a 12-well plate at a density of 600 cells/well. The culture medium was changed every 3 days. Two weeks later, the plated cells were washed twice with phosphate-buffered saline and then treated with 4% polyformaldehyde for 10 min, stained with 0.1% crystal violet for 10 min, and washed with double-distilled water 3 times. Photos of the culture surfaces on dried plates were taken. For a (3-(4,5-dimethylthiazolyl-2)-2,5-diphenyltetrazolium bromide (MTT) proliferation assay, MDA-MB-231 cells (3 × 10^3^/well) were seeded in a 96-well plate. The cell proliferation rate was determined by measuring the absorbance of MTT according to the manufacturer’s instructions (Thermo, USA) at 24 h, 48 h, and 72 h. A microplate spectrophotometer (BioTek, Winooski, VT, USA) was used to measure the absorbance of each sample at 490 nm.

### Cell migration and invasion assays

Wound-healing and Transwell assays were performed to evaluate the migration and invasion of MDA-MB-231 cells transfected with miR-200b-3p or miR-429-5p mimics (NC mimics as the control). The cells were cultured as described in section 2.1 and when the cells reached 90% confluence, the plate containing the cells was scratched with a 200-μL pipette tip. Photos of the cells in culture were taken at 0 and 24 h from the same position to assess cell motility. Cell migration and invasion were also evaluated using Transwell assays with or without Matrigel. After MDA-MB-231 cells’ treatment with miR-200b-3p mimics, miR-429-5p mimics, or NC mimics, 5 × 10^4^ cells/well were seeded in the upper chambers of a 24-well Transwell plate (Corning, NY, USA) with 200 μL of DMEM supplemented with 0.1% bovine serum albumin. The lower chambers were filled with 600 μL of DMEM with 10% bovine serum albumin. The cells were cultured for 12 h to 16 h, and then the outer surface of each insert was washed 3 times with PBST, fixed with 4% polyformaldehyde, and stained with 0.1% crystal violet for 10 min. Photos of the bottoms of the inserts were captured when the surface had dried.

### Quantitative reverse transcription-polymerase chain reaction

MDA-MB-231 and HCC1937 TNBC cells were treated with miR-200b-3p mimics, miR-429-5p mimics, or NC mimics in 6-well plates. The cells were collected 36 h later for total RNA isolation with TRIzol reagent (Invitrogen) according to the manufacturer’s instructions, and cDNA was generated using a real-time polymerase chain reaction (PCR) kit (Takara, Shiga, Japan). Quantitative reverse-transcription polymerase chain reaction (qRT-PCR) was performed on a 7900HT Fast real-time PCR system (Applied Biosystems, Singapore). We followed our previously published amplification procedure: 5 min at 95°C, followed by 40 cycles at 95°C for 30 s and 65°C for 45 s [14]. Expression of mRNA was assessed by evaluating threshold cycle values. The threshold cycle values of *LIMK1* were normalized to the level of GAPDH expression. The primer sequences used were as follows: LIMK1-forward: 5′-CAAGGGACTGGTTATGGTGGC-3′; LIMK1-reverse: 5′-CCCCGTCACCGATAAAGGTC-3′; GAPDH-forward: 5′-CATGAGAAGTATGACAACAGCCT-3′; and GAPDH-reverse: 5′-AGTCCTTCCACGATACCAAAGT-3′. The qRT-PCR results were analyzed using the 2^−∆∆t^ method [42].

### Western blot analysis

MDA-MB-231 and HCC1937 TNBC cells were collected after 72 h of treatment with miR-200b-3p, miR-429-5p and NC mimics, and total protein samples were extracted from them using a RIPA buffer (Beyotime Biotechnology, Shanghai, China). Protein concentrations of the samples were measured with a BCA Protein Assay Kit (Beyotime Biotechnology) according to the manufacturer’s instructions. The protein samples (60 μg) were denatured with 6× sodium dodecyl sulfate (SDS) loading buffer (100 mM/L Tris-HCl, pH 6.8, 4% SDS, 0.2% bromophenol blue, 20% glycerin, 200 mM/L β-mercaptoethanol) at 95°C for 5 min. The denatured protein samples were separated on a 10% SDS polyacrylamide gel using electrophoresis and transferred onto 0.45-μm nitrocellulose membranes (Beyotime Biotechnology). The membranes were blocked for 60 min with a 5% nonfat milk solution and then incubated with primary antibodies overnight at 4°C. The blots were washed with PBST and incubated for 1 h with an anti-rabbit or anti-mouse secondary antibody (1:2000; Santa Cruz Biotechnology, Santa Cruz, CA, USA). After 3 washes with phosphate-buffered saline with Tween 20, immunoreactive protein bands were detected using an Odyssey Scanning System (LI-COR Biosciences, Lincoln, NE, USA). Primary antibodies against the following were used: PCNA (1:2000; Cell Signaling Technology, Danvers, MA, USA), MMP2 (1:1000; Arigo Biolaboratories, Hsinchu City, Taiwan, China), MMP9 (1:1000; Arigo Biolaboratories), LIMK1 (1:500; Arigo Biolaboratories), CFL1 (1:500; Arigo Biolaboratories), phospho-CFL1 (1:500; Arigo Biolaboratories), CDK2 (1:2000; Bioworld, Shanghai, China), CDK4 (1:2000; Abcam, Cambridge, MA, USA), CDK6 (1:2000; Abcam), cyclin D1 (1:10 000; Abcam), cyclin E1 (1:2000; Abcam), and β-actin (1:2000; Santa Cruz Biotechnology, CA, USA).

### Cell cycle assay

MDA-MB-231 cells were treated with miR-200b-3p mimics, miR-429-5p mimics, or NC mimics for 36 h. The cells were then collected, washed, suspended in phosphate-buffered saline, and fixed in cold 70% ethanol overnight at 4°C. After a 30-min digestion with RNase (0.1 g/L), 300 μL (0.05 g/L) of propidium iodide staining solution was added to each cell sample. The stained samples were incubated for 30 min at room temperature in the dark. The cell-cycle progression was then analyzed using flow cytometry.

### Dual-luciferase reporter assay

HEK 293T cells were seeded in 48-well plates and cultured until they reached 70% confluence. The psiCHECK-2/LIMK1 3′-untranslated region (UTR) and psiCHECK-2/LIMK1 3′-UTR mutant reporter plasmids were purchased from Ibsbio. HEK 293T cells were transiently cotransfected with 0.2 µg psiCHECK-2/LIMK1 3′-UTR or psiCHECK-2/LIMK1 3′-UTR mutant reporter plasmids together with 100 nM/L miR-200b-3p mimics, miR-429-5p mimics, or NC mimics using Lipofectamine 2000 according to the manufacturer’s instructions. After 72 h, cell lysates were collected to determine firefly and Renilla luciferase activity using a Dual Luciferase Assay Kit (Beyotime Biotechnology). Firefly luciferase activity values were normalized to the values for Renilla luciferase, and the results are presented as the ratio of firefly to Renilla activity values.

### Protein-protein interactions analysis

The protein-protein analysis of related proteins was based on the STRING database (http://string-db.org/). According to the human species in this database, the protein-protein interaction networks among miR-200b-3p, miR-429-5p and LIMK1-CFL1 pathway were built by extracting the related genes from the database, and then the networks were built.

### Statistical analysis

Data from at least 3 separate experiments are presented as means ± standard errors of the mean. The Student *t* test and one-way ANOVA were also performed to compare the difference. Differences were considered significantly at *p* values less than 0.05. GraphPad Prism software version 6.0 (GraphPad, San Diego, CA, USA) was used for all statistical analyses.

## SUPPLEMENTARY MATERIALS TABLE



## Abbreviations

TNBC: Triple-negative breast cancer
MiRNAs: MicroRNAs
LIMK1: LIM domain kinase 1
CFL1: cofilin 1
BC: Breast cancer
IKBKB/IKK-β: nuclear factor-kappa B kinase subunit beta
ZEB1: zinc finger E-box binding homeobox 1
CRKL: CRK-like proto-oncogene, adaptor protein
XIAP: X-linked inhibitor of apoptosis
MMP: metalloproteinases
SP1: Sp1 transcription factor
53BP1: p53 binding protein 1
RHOGDI: Rho GDP disassociation inhibitor alpha
DMEM: Dulbecco’s modified Eagle’s medium
NC: negative-control
MTT (3-(4,5-dimethylthiazolyl-2)-2: 5-diphenyltetrazolium bromide
PCR: polymerase chain reaction
qRT-PCR: Quantitative reverse-transcription polymerase chain reaction
SDS: sodium dodecyl sulfate
