# Supplementary material for: The microRNAs miR-200b-3p and miR-429-5p target the LIMK1/CFL1 pathway to inhibit growth and motility of breast cancer cells
Source: Oncotarget. 2017 Jul 12;8(49):85276–89. doi: 10.18632/oncotarget.19205 (PMC5689609; doi:10.18632/oncotarget.19205)
Supplement: Supplementary file 1 [file oncotarget-08-85276-s001.pdf]

The microRNAs miR-200b-3p and miR-429-5p target the LIMK1/CFL1 pathway to inhibit growth and motility of breast cancer cells

SUPPLEMENTARY MATERIALS

Supplementary Table 1: Expression of miR-200b-3p and miR-429-5p in breast cancer tissue and cell lines, based on publications in PubMed

Study PMID	Sample type	MiR-200b-3p expression	MiR-429-5p expression
22964023	Tissue	Low	-
23372687	Tissue	High	-
25639535	Tissue	Low	-
26201425	Tissue	Low	-
24447584	Tissue	Low	Low
26062653	Cell line	Low	Low
18376396	Cell line	Low	Low
20390345	Cell line	Low	-
22562546	Cell line	Low	-
24447584	Cell line	Low	-
24925028	Cell line	Low	-
25886595	Cell line	Low	-
25639535	Cell line	Low	-

The search terms were “miR-200b AND breast” or “miR-429 AND breast” in PubMed database. The expression of miR-200b-3p and miR-429-5p was calculated individually based on each cited publication.
